# Reviewers in 2018

**DOI:** 10.1098/rsos.190238

**Published:** 2019-03-06

**Authors:** Jeremy K. M. Sanders

**Affiliations:** Editor-in-Chief

The Editors of *Royal Society Open Science* would like to thank all reviewers who contributed their time and expertise by refereeing manuscripts submitted throughout 2018. From previous feedback, we know that most reviewers value recognition of their efforts by the journal and through services such as *Publons* which are integrated with *Royal Society Open Science*.

To provide an opportunity for recognition, we list the names of all reviewers (unless they have opted out). This article is made permanent and citeable by its digital object identifier (DOI). We encourage you to quote your contribution in your applications for tenure, promotion and grants, or other forms of assessment. Where you have provided it, we have included your ORCID, so your contribution can be unambiguously assigned to you.

The team greatly appreciate all the work our reviewers do on behalf of the journal, and we look forward to working with you again in 2019.

Dentz M

Whitney F

Ríos Chelén A

Stockhausen W http://orcid.org/0000-0003-3633-2157

Li H

Sachdev A

Muthusamy S

Cubillos J

Xue Z

Blumstein D http://orcid.org/0000-0001-5793-9244

Wilkinson F

Murthy YVVS

Fordyce RE

Borregaard M

Carpenter G

Green J

Phan R

Waters A

Liu Y http://orcid.org/0000-0003-1676-4446

Dhaliwal GS

Wu X

Weihmann T http://orcid.org/0000-0002-6628-1816

Huang M

Smith M http://orcid.org/0000-0001-5660-1727

Ellis S http://orcid.org/0000-0001-9019-6040

Saravanan R http://orcid.org/0000-0002-3771-4694

Wichmann S

Sutton G

Micheli D

Scaglion R

Overcast I

Ruan J

Sun X

Ali E

Xu W

Iacolina L

Moon B

Steinbauer MJ

Linko V

Benton M http://orcid.org/0000-0002-4323-1824

Morrill L

Dinovitser A

Scelza B http://orcid.org/0000-0001-5875-8875

Frank S http://orcid.org/0000-0001-7348-7794

Suleiman R http://orcid.org/0000-0001-9848-7475

Riebel K http://orcid.org/0000-0003-2373-8510

Jasim D

Bromage T

Worthy TH http://orcid.org/0000-0001-7047-4680

Wang D

Lion S http://orcid.org/0000-0002-4081-0038

Baddeley R

El Naschie M

Rosenblatt N

Tzou Y-M http://orcid.org/0000-0002-3680-7303

Feller K

Ge M

Campo A http://orcid.org/0000-0003-4817-4790

Evano G

Warburton N

Taniguchi I

Hancean M-G

Brooks M

Oh H

Schuster S http://orcid.org/0000-0003-2828-9355

Booth W

Al-khalili J http://orcid.org/0000-0003-3181-5280

Russo I-R http://orcid.org/0000-0001-9504-3633

DeDeo S http://orcid.org/0000-0002-5346-9393

Haider S http://orcid.org/0000-0002-1738-6945

Lambert M http://orcid.org/0000-0003-3618-7260

Omachi H

Harris T

Kalcounis-Rueppell M

Hui J http://orcid.org/0000-0003-1355-8495

Abdou E

Hall M

Mavri J

Cummins F

Paillusson F

Guler MO http://orcid.org/0000-0003-1168-202X

Giarra M

Orr P

Rocca I

Elbaum M

Tan X

Bibi N

Kara HE

Mazzoni R

Liu W

Pilakouta N http://orcid.org/0000-0001-8503-520X

Hedenström A http://orcid.org/0000-0002-1757-0945

Fernández-Lafuente R

Yan J-K

Kim H-T

Freitak D

Wang L-X

Jiang G

Zajitschek S http://orcid.org/0000-0003-4676-9950

Qiu J

Xie L

Kalckert A

Bujorianu M

Cheng Y

Martinez-Camblor P

Tougard C

Ortega-Hernández J http://orcid.org/0000-0002-6801-7373

Yan C

Hauber ME http://orcid.org/0000-0003-2014-4928

Jensen P

Bernabeu M

Somero G

Johnson VE http://orcid.org/0000-0002-8659-4772

Mehdi-Rashidi M

Fu Y

Indurkhya B

Nikora V

Parry L http://orcid.org/0000-0002-3910-0346

Li D

Albers C

Hu Z

Bai L

Zhang J

Bulgariu L

Wu J-M

Hu L

Dai L

Lei B

Chan T

Costine A

Cuadrado C

Beutel R

Miall C

Dhinojwala A

Huang W http://orcid.org/0000-0003-4069-7343

Bianucci G http://orcid.org/0000-0001-7105-0863

Emslie S http://orcid.org/0000-0001-9112-5690

Di Maria F

Werth A http://orcid.org/0000-0002-7777-478X

Cao R

Bell J

Rojas B http://orcid.org/0000-0002-6715-7294

Saramago B

Fontoura N

Kerr R

Gao X

Kadmi Y

Avramopoulos A

Harvey T http://orcid.org/0000-0002-2717-7004

Papadakis R http://orcid.org/0000-0001-9734-7550

Stayton T

Litton CD

Ready P

Zhang S

Wrangham R

Mori E

Lintao Z

Yu Z http://orcid.org/0000-0002-0209-7884

Cheng X

Li J http://orcid.org/0000-0003-2324-0501

Maenosono S

D'Ausilio A

McCarthy M

Berthaume M

Houssaye A

Harikumar R

Wieczorek S

Zeder M http://orcid.org/0000-0001-9716-4877

Elimelech Y

Croston R

Nonacs P http://orcid.org/0000-0003-3010-0777

Zhang S-F

Clark A http://orcid.org/0000-0001-5908-2862

He X

Xia Y

Hagimori M

Kumar N

Cavin L

Carlen ET

Harbach R http://orcid.org/0000-0003-1384-6972

Patra A

Jungwirth A

Grabowska B

Krishnan S

Anbarasan P

Root-Bernstein M

Reyes-García V

Kendal W http://orcid.org/0000-0003-4054-4312

Frank T http://orcid.org/0000-0002-9329-2414

Han B

Yu H

Manzhos S

Prasad G

Summerhayes G

Mohan S

Hufnagel L

Ehret A http://orcid.org/0000-0002-8740-8900

Holmes N

Weisbecker V

Sato T

Maridet O

Toyoma T

Du L

Eberini I

Bowman V

Browne D

Coscia M http://orcid.org/0000-0001-5984-5137

Xue BK

Ye J

Li X

Boomsma JJ

Antonioni A http://orcid.org/0000-0002-5788-3348

Hutchinson J

Vervoort M

Lameris T

Miniaci M

Wilkerson R

Ghalambor C

Fitzgerald E

Kumar S

Spehar B

Kotabe HB

DeLong J http://orcid.org/0000-0003-0558-8213

Briggs D http://orcid.org/0000-0003-0649-6417

Garab G

Gleason J

Sorrentino A http://orcid.org/0000-0003-1048-3002

Li Y

O'Brien M

Bosia F http://orcid.org/0000-0002-2886-4519

Wang G-J http://orcid.org/0000-0002-2813-4356

Frere C http://orcid.org/0000-0002-9671-2138

Lima F

Thuesen E

Martínez-Castelao A

Yao Y

Stroustrup N

Nikolic N

Paulevé L

Lameira A http://orcid.org/0000-0003-3071-4824

Lempidakis E http://orcid.org/0000-0003-2384-9093

Roose B

Casey E

Alvarez-Buylla E

Oliveira A

Sommer W

Marx F http://orcid.org/0000-0002-1029-4001

Agarkar SA

Nielsen A

Jurado-Sanchez B

Wang GG

van de Water J

Tang J

Dai K

Verma P

John B

Akhavan O

Genty E

Nakano R

Meinhardt-Injac B

Stark A http://orcid.org/0000-0002-4217-2850

Xin L http://orcid.org/0000-0002-4683-4612

Lambiotte R

Ying Y http://orcid.org/0000-0002-3867-5893

Viré A

East M

Zhang Y

Rahnev D

Schmitz J

Sedaghat A

Kushner D

Choiniere J

Tan R

Ellis R http://orcid.org/0000-0002-3117-0075

Ciaccio E

Nolin D

Wiktor S

Anindya S

Robinson N

Li Y

Endler J http://orcid.org/0000-0002-7557-7627

Duong DL http://orcid.org/0000-0002-4118-9589

Riegel H

Chou I-C

Lekgoathi M

Favier L

Badola R

O'Cearbhaill ED

Dong W

Ma X

Liang X

Her S

Blair R

Qi R

Polly P

Heath S http://orcid.org/0000-0002-9822-8091

Wang T

Noren J

Das B

Karaagac H

Fan Y

Vega V

Plön S

Uroos M

Bracken-Grissom H

Fay L

Martin-Galvez D http://orcid.org/0000-0002-1501-9578

Dzyuba PSV

Becker D http://orcid.org/0000-0003-4315-8628

Drzewiecka K

Shi X

Bowers D

Loch C

Baños R http://orcid.org/0000-0001-7844-4902

Wee BS http://orcid.org/0000-0002-6745-9727

Noë R

van der Burg E

Russo D

Zhao Y

Moodley B

Delivopoulos E

Zebitz CW

Meunier F

Sedev R

Zhang Y

Tie Y

Molnar J

Zhao X

Kuijper B http://orcid.org/0000-0002-7263-2846

Smith S

Nuijten M

Colquhoun D http://orcid.org/0000-0002-4263-017X

Ziker J

Wang D

Bhattacharjee S

Lages J

Boyde A

Rodrigues T

Koszytkowska-Stawińska M

Lakes-Harlan R

Rawlence N http://orcid.org/0000-0001-6968-6643

Han J

Tabata J http://orcid.org/0000-0001-9095-2694

Shikov A

Lee P

Sciani J http://orcid.org/0000-0001-7213-206X

Buccianti A

Krings M

Quintana M http://orcid.org/0000-0002-7036-8658

Ingegno B

Lusher A

Foster K

Ahmad N http://orcid.org/0000-0001-5067-7368

Smolska E

Koizumi H http://orcid.org/0000-0001-9225-7089

Yin J

Geisler J

Gu C

Kumar S

Hershkovitz I

Xu Y-J

Magnotti J http://orcid.org/0000-0003-2093-0603

Shelton D

Ge P

Paluh D

Zingales B

Skoulikaris C

Seenivasan S

Khatri V

Aiello L

Deeming DC http://orcid.org/0000-0002-9587-6149

Bradley D

Nalam P

Ngonghala C

Chunming S

Bowen B

Matabos M

Rowland DR

Gökoğlu G

Mitoraj MP

Lucas C

Waldman B http://orcid.org/0000-0003-0006-5333

Wang S

Hegedus R

Leslie D

van der Hoek Y

Campbell M

Boyd R

James H

Gremillion K

Power E

Mu T

Qian L

Stanley E

Holton G

Lammers M http://orcid.org/0000-0002-7215-4426

Jing Z

Jingchen Z http://orcid.org/0000-0003-3132-916X

Llobet E

Maw M http://orcid.org/0000-0001-8661-1707

Rako N

Brancher P

Van Der Molen S

Peterson S

Zhang JJ

Skulmowski A

Cox B

Storrs K

Churchill M

Wei J

Lopman B

Patterson R

Michelini E

Zaburdaev V

Vijayan KK

Sharma S

Yang Y

Bourikas K

Pánek M

Qi JL

Arcaute E

Tammaru T http://orcid.org/0000-0002-6892-5910

Kessinger T

Stockin K

Creel S

Sallan L

Wang L

Wiley J

Zhu W

Vargas-Bernal R http://orcid.org/0000-0003-4865-4575

Gane E

Akcay E http://orcid.org/0000-0001-8149-7124

Yu L

Wang F

Clark C http://orcid.org/0000-0002-7692-8150

Kessler V

D'Abramo G

Hufschmid J

Requena JM

Ma C

Breck J

Macy M

Culbertson C

Kalinkat G

Yuan Z

Byng JW

Pal A

Yovel Y

Liebchen B

Figueirido Castillo FB http://orcid.org/0000-0003-2542-3977

Serre D

Grillo A

Rajagopal S

Balanay M

Thakur VK

Pasetto M

Frainer A

Petrakis P

Piscia R

Taju G

Turner J

Li J

Saleem A

Santiago J

Eng C http://orcid.org/0000-0002-1081-6463

Trevizan B

Alberto V

Woodall L

Clementz M

Wolf C http://orcid.org/0000-0002-5519-3845

Kadar C

Velez-Juarbe J

Buckeridge J

Dong G

Zhang D

Rajamanickam A

van Grunsven R

Mushayabasa S http://orcid.org/0000-0002-7423-2934

Eder M

Mallow O

Busca G

Li C

Voss H

Vergani L http://orcid.org/0000-0003-2353-7751

Hardman S

Liu J

Dzialowski A

Peng Y

Guo Y

Liu X

Feng G

Vasam C

Fan X http://orcid.org/0000-0002-9039-6736

Faria G http://orcid.org/0000-0002-1511-8680

Goldenberg S http://orcid.org/0000-0002-9468-8920

Villnas A

Rees H

Simpson AM

Kamarehie B

Zoumpoulakis P

Saperstein A

Wang Q

Jähne B

Liu X

Gentry K

Von Lukas UF

Kabbara J

Yang H

Federle W http://orcid.org/0000-0002-6375-3005

Currie T

Cao Y http://orcid.org/0000-0002-4953-8886

Ji Y http://orcid.org/0000-0002-4604-4258

Bach P

Li H

Vogt T

Honer Zu Siederdissen C

Conte P

Niu J

Herrera López E

Brightsmith D

Mutlugun E

Liew KW

Bonacin J

Dunbar R http://orcid.org/0000-0002-9982-9702

Stocker A

Hussain M

Schumann D

Li J

Jaafari J http://orcid.org/0000-0002-0186-7779

Mayer-Gall T

Li W

Sbardella G

Han TA

Cadena E

Chen H

Rondoni G

Duman G

Li W http://orcid.org/0000-0001-7801-3643

Sturdy C

Li J

Ruiz de Miras J

Wang X-F

Huang T

Wang X

Li Q

Le Borgne M

Stoyanova R

Gjesfjeld E

Luo RF

Li Z

Carey D

Sinniah K

De Lillo C

Knapp R http://orcid.org/0000-0001-6923-8410

Li JA

Rajarathnam D

Nizami A-S

Mehta V

Fathima N

Ferguson HJ

Pacultová K

Thompson AK

Bruder A

Arcibar Orozco JA

Geitner N

Mukherjee S

Naushad M

Stewart A

Boessenecker R http://orcid.org/0000-0002-6117-128X

Wang Q

Bolsterlee B

Dugas M

Greenebaum B

Meunier J http://orcid.org/0000-0001-6893-2064

Bentley A

Hancock P

Dunn K

Whalen M

Gorissen M

Kumar G

Buckley C

Rezaei M

Tseng J http://orcid.org/0000-0001-5335-4230

Quintana S

Bub G

Kolasinski KW

Sarles S

Dzhumanov S

Kuroda K

Singh B

Romano L

Nur H

Swaney W

Butler M

White S

Barletta A http://orcid.org/0000-0002-6994-5585

El-Shishtawy R

Leger L

Trippier P

Brennan R http://orcid.org/0000-0001-7678-564X

Fan G

Liu J

Taniguchi N

Pop I

Kumara HN

Faith T

Szablowski PJ

Ojaghi M

Van Liedekerke P

Kvifte G

Tanaka Y

Lonzarich G

Donelson J http://orcid.org/0000-0002-0039-5300

Evans A

Riziotis C

Crofts J

Xiong H

Lopez-Bucio J

Li Z http://orcid.org/0000-0002-3230-5955

Zhu J

Zamin T

Liao Q

Gruber T http://orcid.org/0000-0002-6766-3947

Yamashita A

Prashantha K

Capelluto DGS

Kim YA

Xia C-Y

Cao X

Hamid S

Jin M

von Byern J

Gupta HS http://orcid.org/0000-0003-2201-8933

Dunlop R http://orcid.org/0000-0002-0427-6317

Yestrebsky C

Yu F

Yu Y

Mahdi F

Uggla C http://orcid.org/0000-0003-1639-3307

Diana G

Kilbourne B

Yavuz M http://orcid.org/0000-0003-3452-8551

Croatt M

Harris E

Gebeshuber I

Georgiev A

Farine D http://orcid.org/0000-0003-2208-7613

Tobin A

Douhard M http://orcid.org/0000-0001-9422-7270

Michiels N http://orcid.org/0000-0001-9873-3111

Cipora K

Erba M http://orcid.org/0000-0002-2172-592X

Jiang C

Ha K

Hernandez P

Finch T

Pal M

Gumert M

Verboord M

Vinther J http://orcid.org/0000-0002-3584-9616

Crockett KA

Senn S

Debnath SM

Jesenik M

Gao X http://orcid.org/0000-0003-2101-1609

Seibel B

Shi X

Sarkar S

Hein A

Fernandez-Juricic E

Yang J http://orcid.org/0000-0001-5934-1537

Zhang H http://orcid.org/0000-0002-1716-5763

Jin Z

Elliott K http://orcid.org/0000-0001-5304-3993

Zhu J

Sun Y

Rørvang MV http://orcid.org/0000-0002-3503-2059

McElligott A

Jaiswal S

Long Y

Bao X

Jiang X

Gomez-Lievano A http://orcid.org/0000-0001-8320-0857

Caputi N

Mitrovic M

Nugroho LE

Bulfin B

Chu F

Chin A

Li B

Song N

Vincent B

Jones B http://orcid.org/0000-0001-7777-0220

Matzel L http://orcid.org/0000-0003-0462-7188

Pidko E

Huang W-J

Pappas D

Erdem E

Yang C

Late D

Lazzara G http://orcid.org/0000-0003-1953-5817

Lambert S

Fiems D

Song Y

Bjerrum-Bohr NEJ

Perinelli D

Riyahi S

Lopez JM

Barot S

Gaur PK

Qayyum MF

Moreno C

Warpeha KM

Moore M http://orcid.org/0000-0002-8255-4247

Zhao W

Skowronska A

Martins L

Shaw R

Nabae Y

Kittakoop P

Takeuchi H

Wei G

Favre-Réguillon A

Ahlberg P

Brierley I

Xiao Y

Wiklund H

De Micheli M

Filippi P http://orcid.org/0000-0002-2990-9344

Green R

Cayuela L

Ma S

Podobnik B

Vaccaro C

Pang X http://orcid.org/0000-0003-2445-5221

Kang C

Doi H http://orcid.org/0000-0002-2701-3982

Drewe J

Grimes DR http://orcid.org/0000-0003-3140-3278

Luo J

Gopalakrishnan A

Schönfeld L-M

Anné J http://orcid.org/0000-0002-6838-4317

Planat M http://orcid.org/0000-0001-5739-546X

Goder R

Imhof H

Fakhrullin R http://orcid.org/0000-0003-2015-7649

Chappell M

Gao B

Basirun WJ

Dorozhkin S

Bilic I

Gómez-Benito MJ

Nguyen H

Costa-Boeddeker S

Kitson D

Correia I

Moreno-Tost R

Schaefer J

Witteveen B

Cameron E

Wojnicz D

Luo F

Gu J

Henderson A http://orcid.org/0000-0002-5944-3135

Aravind NA

Dalvi S

Estellé P

Purkait T

van Wingerden M

Maclaren I

Dias I

Biondi A

Bindemann M

Laine E

Deng G-J

Vuong K

Yamamoto T

Pitsikalis M

Geard N

Pietrowska-Borek M

Binks B

Pintor L

Arenas-Mena C

Zhang D

Carabineiro SAC

Albiach Serrano A

Kościński K

Thompson D

Zhang Y

Ou R

Tieri G

Huth M

Trumbo S

Neda Z

Ghorbani-Vaghei R

Bao X

Yu W

Zerbini A

Gindl W http://orcid.org/0000-0002-8224-6762

Lu Y

Pan Y

Martius C

Xiang Q

Zhang X

Hu H

Feinberg D

Guagliardi I

Joyce S

Liu X

Stephen I

Read de Alaniz J

Pang DJ http://orcid.org/0000-0001-6965-4166

Dong Y

Abid M

Pucci A

Gao F

Giles S http://orcid.org/0000-0001-9267-4392

Johan E

Jain G

Ghosh S

Henderson L

Ngo HV

Hoshi A

Wermelinger S

Bertram J

Wang J

Hoch M

Aceituno F

Jussim L

Zhang L

Kulkarni PP

Takahashi K

Altun NE

Chen FX

Moon B

Wang Y

Tello-Aburto R

Ziegler A

Colman E

Kroliczak G

Shu R

Carey JW

Stewart J

Omotosho OE

Onyshchenko I

Gabard G

Agrawal A

Pan Y

Zhang T

Li T

Lu Z

Yi-Tao L

Walton JC

Dogra A

Hughes C

Alves CR

Orłowski G

Aguirre JD

Hamlin K

Strader M

Umemura M

Tuberty S

Torres-Sánchez J

Pleimling M

Zhang S

Panigrahi P

Wu J

O'Neil G

Snively E

Lima DB

Chen D

Li X

Kan T http://orcid.org/0000-0002-8468-4475

Szeleszczuk Ł

Adamatzky A http://orcid.org/0000-0003-1073-2662

Zhang Q

Singh AK

Liu M

Mishra S

Goniewicz K http://orcid.org/0000-0003-4368-6850

Lobaskin V

Bao Y

Dominy N http://orcid.org/0000-0001-5916-418X

Sarasua JR

Schwing R http://orcid.org/0000-0003-4663-8432

Eberl J-M

Nadolny K

Grow A

Zhu L

Luo S

Seybold S

Vasilescu A

Özkir D

Nicolini P

Yuan Y

Wu X

Mukhopadhyay SC

Tria F

Vermant J

Huang Y

Clark C

Adams M

Jacob J

Dorai K

Moldoveanu F

Krishna LR

Hanna J

Gasparrini F

Stambuk BU

Nami H

Geiser F

Narayan A

Song K

Yin X

Bose N

O'Hara M

Samui A

Wagenmakers E-J

Mohanty AK

Rajendra K

Bertails-Descoubes F http://orcid.org/0000-0001-8930-3048

Tassoulas J

Reeves A

Vinauger C

Gow E

Xu Y http://orcid.org/0000-0002-7962-6668

Huo F

Hippler M

Gentili R

Bianca C

Spoelstra K http://orcid.org/0000-0001-8614-4387

Garcia-Prada JC

Manayi A

Ino K

Zhang Y

de Klerk C http://orcid.org/0000-0002-6005-7880

Yin S

Mukherjee A

Sfakianakis N

Jasuja K

Pomerleau C

Feng C

Hu J

Wei J

Saraf S

López M

Xu B

Outlaw D

Simonyan K

Brevik I

Shao Q

Chem L

Kim C http://orcid.org/0000-0002-7580-0374

AlMayouf AM

Bhagavatula P http://orcid.org/0000-0002-3115-0736

de Carvalho M

Kramers J

Civale J

Tonga G

Zheng H

Zhang J

Cárdenas J

Field K http://orcid.org/0000-0002-5196-2360

Rout CS

Srinivasa Rao S http://orcid.org/0000-0002-1308-7067

Dahanukar N

Bernstein D

Marmo F

Heritage J http://orcid.org/0000-0002-8603-6447

Ambike S

Harper J

Wang L

Okazaki T http://orcid.org/0000-0002-5958-0148

Penta R

Ruff C

Reid TW

Okabe S

Tsuji T

Harasti D http://orcid.org/0000-0002-2851-9838

Kristoufek L

Li L

Armbruster D http://orcid.org/0000-0001-9681-9173

Vaidheeswaran A

Geng H-Z http://orcid.org/0000-0001-7897-8584

Lenau T

Villa I

Mohammed BJ

Hwang DS

Kaburu S http://orcid.org/0000-0001-7456-3269

Kubinyi M

Xu C

Prasad K

Liu X http://orcid.org/0000-0002-2299-9279

Oziolor E

Vedrine J

Peng J

Wang Y

Quan D

Lianos P

Soares R

Willems W

Abdelrahman E

Evans M

Konarev D

Cheshire M

Thompson S

Takabayashi Y

Sobotka T

Murcio R

Jia X

Okamura H

Wang C

Cameron P

Rose M

Sun G-Q

Liu Y

Boz MA

Burris D

Laia CAT

Paquet M http://orcid.org/0000-0003-1182-2299

Wojcieszak R

Vidoni R

Brouwer A http://orcid.org/0000-0002-3779-5287

Syndercombe Court D

Pereira D

Capriotta AL

Zou M

Li S

Figner B

Borstnar R

Dunham Y

Latanowicz L

Fei Z

Jones N http://orcid.org/0000-0002-6031-7507

Silverman E http://orcid.org/0000-0003-0147-6118

Farooq RK

Zhang F http://orcid.org/0000-0001-6348-5816

Feng JJ

Melià P

Gróf G

Sarmento-Soares LM

Caginalp C http://orcid.org/0000-0002-3105-3952

Talwar S

Guo T

Kawai T

Luo Z-X

Chen H

Campos-Montoel RG

Coltro WKT

Shi S http://orcid.org/0000-0001-8984-9809

Harris P

Ma L

Kabir S

McBurney P

Savolainen O

Carrascal L

Crisponi G http://orcid.org/0000-0002-3902-0231

Kumar S

Demiris N

Ebersole C

Jiao T

Wang J-P http://orcid.org/0000-0001-7082-8864

Fu S-J

Açıkalın K

Hoult NA

Wang J

Johnson E

Qin Y-Y

Plaza L

Fu S

Ju Y

Linclau B

Ma X

Vilar M

Scotch M

Wang S-F

Lorenzi A

Ebrahimi A

Pibars S

Zhou J

McMenamin M http://orcid.org/0000-0002-3764-0963

Petterson L

Di Iorio L

Mobashsher AT

Chatterjee K http://orcid.org/0000-0002-7204-2926

Rana K

Giudetti A http://orcid.org/0000-0002-9204-040X

Ergüder Bayramoğlu TH

Dushoff J http://orcid.org/0000-0003-0506-4794

Longrich N

Grogan K

Ben Ishai P http://orcid.org/0000-0001-7394-019X

Acien G

Harrison D

Ayyash M

Shan G

van Assen JJR

Troisi C http://orcid.org/0000-0002-4036-3848

Montevecchi W

Buchard A

Urry H

Barreiro A http://orcid.org/0000-0003-0286-4139

Mohammadi G

Oliveri V

Alexander M

Kis A http://orcid.org/0000-0001-8728-4795

Hall R

Tejero J

Yazdani-Chamzini A

Rodriguez-Fernandez DE

Skeldon A

Eritja R

Feteira A

Teodoriu HC

Valiyaveettil S

Ochowiak M

Nataraj D

Pinto D

Nishide Y http://orcid.org/0000-0002-5978-3367

Karayiannis N

Wei H

Römer D

Zahedi A

Treiber M

Arnold D

Kallio S

Hassim A

Hugel T

Malucelli G

Zhang Q

James SC

Zuo Y

Johnson M

Mikuma T

Xiao W

Wei FH

Potts J http://orcid.org/0000-0002-8564-2904

Chen C-T

Zhao F

Wang J

Shaaban K

Colangelo F

Basarir F

Deshpande A

Filep JG

Whittaker DG

Ni D

Meier R http://orcid.org/0000-0002-4452-2885

Andersen K http://orcid.org/0000-0002-8478-3430

Paquay S

Dudley R

Zhang N

Wang B

Werner D

Goutcher R

Sabariswaran K

Shan ZB

Krause M

Ewers B

Kemper C http://orcid.org/0000-0002-4452-8259

Ni G

Morandini J

Wang P

You Z

Sahiner N

Beisner B

Zhang Y-X

Crowther M

Manoharan S http://orcid.org/0000-0003-4958-4185

Goldingay RL

Balas B

Vityuk A

Da Silva Camargo R

Morin P

Ahmed ZT

Ma KY http://orcid.org/0000-0001-5690-2319

Welcomme R

Akkurt M

Love A

Shi X

Jiang C http://orcid.org/0000-0003-0520-9754

Zhou W

Portet S

Zhang R-b

Ng KC

Dubey KV

Reddy MS

Zhu L

Sheehan M http://orcid.org/0000-0002-3949-7873

Tzourio-Mazoyer N

Evangelista D http://orcid.org/0000-0002-6565-8300

Taheri A

Reeve R

Zhang X

Peng T

Sahoo SK

Gotanda K http://orcid.org/0000-0002-3666-0700

Stafford K http://orcid.org/0000-0003-0039-5025

McCowan B

Martinez L

Lu J

Cuomo S

Saçmacı Ş

Omori R

Morrow J

Nagy M

Yang C

Schwameder H

Rainio K

Petter S

Bocedi G http://orcid.org/0000-0002-9131-6670

Stockle M

Li J

Jinyan Z

Dymond M http://orcid.org/0000-0002-9903-4993

Haider H

Shamsoddini R

Solomon A

Wu Y

Byrnes G

Tanaka K http://orcid.org/0000-0001-5584-3521

Makris D

Pavlopoulos T

Chen M

Shcock B

Ruhl S http://orcid.org/0000-0003-3888-4908

Liang H

Matsuyoshi D http://orcid.org/0000-0003-1388-2527

Han Q http://orcid.org/0000-0002-7843-2191

Koldehoff M

Freeman A

Franssen N

He F

Lewis J

Hosseini N

Zangenehmadar Z

Jayasinghe S

Wu L

Seelamantula CS

Zhang H

Dalchau N http://orcid.org/0000-0002-4872-6914

Duchaine B

Arendt D

Gu X

Fan C-Y

Liu E-H

Saini A

Sultan M

Wheeler B http://orcid.org/0000-0002-8478-3385

Park CS

Ponti G

Waller B

Bigoni D

Attia A

Ricketts A

Liang W

Maruyama T

Gu CD

Otero M

Duraisamy C

Rajeswarapalanichamy R

Gao T

Mercado III E

Park HW

Xie SX

Muthamilarasan M

Chabanne H

Rodriguez S

Nan Z

Bate S

Friedman O

Yang Y

Fu J-L http://orcid.org/0000-0002-7667-5755

Bond AD

Yilmaz E

Liang H

Bianchi M

Graves W

Chen Y

Lu Y

Vassallo P

Dong C

Zhao Y-P

Bucar D-K

Gómez-Ruiz S

Bishop P http://orcid.org/0000-0003-2702-0557

Sriramula S

Mehner T

Wang T

Deppe A

Nepomuceno E

Bianchini A

Weippl E

Li B

Niu X

Rousselet G

Bhatnagar Y

Batiste O

McFrederick Q

Marek Z

Kapheim K http://orcid.org/0000-0002-8140-7712

Marasini N

Murall CL http://orcid.org/0000-0002-1543-4501

Brook B http://orcid.org/0000-0002-2491-1517

Guerrero A http://orcid.org/0000-0003-2323-2691

McKey D

Waldmann A

Bie Z

Festa-Bianchet M

Roche B http://orcid.org/0000-0001-7975-4232

Lucena R

Ibsen-Jensen R

Ahmed MU

Dastgir S

Valentini A

Bianchi A

Jue Z

Ruan M

Liu Y

Montell D

Wei R

Zhong C

Vitz J

Reiskind M http://orcid.org/0000-0001-7307-9416

Pollard A

Liu X

Kojio K

Yu H

Cetinkaya AY

Grenning A

Marron S

Kautt A

Demas G

Li Y

Hodgson TL

Slijepcevic P

Garland E

Whytock R http://orcid.org/0000-0002-0127-6071

Wu A

Wang J

Jo H-H

Qu F

Mogulkoc Y

Campos M

Bulley A

Ricciardelli P

Nagashima H

Zhang L

Stein A

Yoshimura J http://orcid.org/0000-0003-1610-1386

Hui HY

Kohno N

Anjanapura R

Iriarte J

El-Gendy N

Ikeda K

Latty T

Wang F

Cimato S

Brassey C http://orcid.org/0000-0002-6552-541X

Yang Q

Legendre P

Nater A

Dunk P

Ajayi E

Rebecca RC

Sills J

Teaford M

Zhu XS

Hu B

Bode S

Norton W

Du Y

Jost J

Mattei TA

Moskat C

Dominiak BC

Beduk F

Auzanneau F

Sebastián-González E

Bergman T

De Sy V

Guerrero Garcia G

Amoroso G

MacLaren J

Bilaloglu S

Wu J

Kaczmarek L

Pennycook G

Paulun V

Milojevic S

Zhang C

Fu J

Luo X

Musciotto F

Mathur M

Tu R http://orcid.org/0000-0002-2697-196X

Zitterbart D

Zaitsau D

Iantovics LB

Samson D http://orcid.org/0000-0003-3318-7652

Quevedo H

Yeom D-H

Jia W http://orcid.org/0000-0001-6242-3269

Karuppiah M

Méndez-Rojas M

Lin X

Bracamonte V

Morin D

MacLean A

Kline M http://orcid.org/0000-0002-1998-6928

Chandler M

D'Auria J

Lopez D

Hileman G

Asgharipour MR

Li H

Zhang J-P

Slavisa J

Jones D

Wu Q

Fernandes V

Martin J http://orcid.org/0000-0001-6100-8581

Schoeberl T

Zhou Y

Jia HJ

Callahan DL

Leskelä M

McDermott C

Garcia D http://orcid.org/0000-0002-2820-9151

Hossie TJ

Hickerson C-A

Reddy MV

Le Pelley ME

He Z

Wang Q

Zhou Q

Quek G

Zhang Q

Gao Y

Tahermansouri H

Yang H

Meng Y

López-Guerrero P

Gomes Rodrigues H http://orcid.org/0000-0002-3667-3296

Nagabhushana H

Huang S

Scheidegger C

Mareschal I

Sharma A

Rosengrave P

Knop E http://orcid.org/0000-0001-9402-2216

Wang J

Sclafani V

Bechelany M

Findlater M

Barak M http://orcid.org/0000-0003-4953-4380

Hidayu AR

Guo Q

Timson D http://orcid.org/0000-0002-0985-8818

Fu D-W

Cheng C

Nadyeina O

Zhang H

Rouabhia M

Shi J-W

Azimirad R

Guan L

Abdelhameed Moustafa NM

Nussey D http://orcid.org/0000-0002-9985-0317

Mylrea M

Wang G-L

Hui KH

Rog T

Kirubakaran S

Cakar P

Ma K-L

Wilson B

Aguilar-Perera A

Wang G

Ma D

Kougias P http://orcid.org/0000-0003-4416-2135

Pickles M

Pimentel L

Nikolic M

Reade H

Wood B

Ker A

Owens H

Agrawal R

Sharma B

Aryal A http://orcid.org/0000-0002-2695-9901

Losytskyy MY

Ahn SJ

Karalis K http://orcid.org/0000-0003-2835-9384

Gomes J

Croissant J

Segarra S

Pomara L

Ma C

Hellmich C

Dai J-J

Shinya M

Honey M http://orcid.org/0000-0001-7272-476X

Li G

Sun M

Moses ME

Norta A

Ganusov V

Kingdom F

Blinov M

Chen G

Alabugin I

Varma R

Ye H-Y

Shen Y

Harrison P

Isse A

Liao S-K

Mathur R

Parker W

Fang X

Abd el-Sataer NA

Liu R

Jones K

Schmidt-Rhaesa A

Palamara GM

Diaz G

Dearden P

Yan X

Mutze G

Bootsma M

Green M

Khayyeri H

Newton I

Rafael R

Georgopanos P

Paulssen H

Kuri-Morales AF

Zhang W

Seethamraju S

Shi Y

Timofeeva M

Majmudar J

Zhang Y

Rowlett P http://orcid.org/0000-0003-1917-7458

Fouad MA

An J

Filingeri D http://orcid.org/0000-0001-5652-395X

Ganesh B

Devarajan A

Lukas D http://orcid.org/0000-0002-7141-3545

Karakashev S

Petersen K

Senju A

Hill E http://orcid.org/0000-0002-2992-2004

Zhang J

Li E

Liu M

Chunxiong B

Subramanian B

Fischer M http://orcid.org/0000-0003-4553-8624

Zhang S

Wilson P

Bräuer A

Zhang G

Smith? R http://orcid.org/0000-0002-6529-3321

Zografopoulos DC

Balazsi G http://orcid.org/0000-0002-6865-5818

Tan A http://orcid.org/0000-0002-9227-4644

Chao B

Wang A

Duong HM

Zhang J

Komarov I

O'Mullane A

Won J-P

Higham D

Reina A http://orcid.org/0000-0003-4745-992X

Coskun A

Wen ZY

Dou M

Kay R

Fang C http://orcid.org/0000-0003-0915-7453

Sun D

English N

Ping J

Nunn C http://orcid.org/0000-0001-9330-2873

Zhao H http://orcid.org/0000-0001-6829-6571

Wu T

Zhang Q

Bajda T

Lipke PN

Hubicki C

Sallam HEM

Wang R

Premasiri WR

Mauro JC

Blyuss K

Macleod M

Kim S-J

Yang F

Rovatti L

Hutchins K http://orcid.org/0000-0001-8792-2830

Bogdanović G

Cheke L

Lonsdorf E

Knapp C

Yildiz A

Blaszczak J

Dykman L

Zhao H

Hawkins J

Hardy I

Ragauskas A

Gou J

Zhang S

Schmitz O http://orcid.org/0000-0003-1515-2667

Sternberg Lewerin S

Codding B

Ottoni E http://orcid.org/0000-0003-1288-8345

Piyasena M

Islam A

Heng L

Sartorius B

Moura A

Woodbury-Smith M

Merino G

Ruusuvuori P

Zhang D

An R

Bauer R http://orcid.org/0000-0002-7268-9359

Arana Alonso S

Liu X

Calisti M http://orcid.org/0000-0002-2590-188X

Hervé A

Siddle H

Cheng L

Brügger R http://orcid.org/0000-0001-8373-2255

Searcy W

Peffer T

Hogg C

Yang Q

Cho JW

Corrales R

Brucato N

Munson B

Jackson B

Sundstrom A http://orcid.org/0000-0002-3378-4513

Pattanayak S

Guo C

Miller R

Lan W

Gillis G

Naji KM

Elwakeel KZ

Clifton G

Wang Y-S http://orcid.org/0000-0002-1774-9494

Aggelis G

Tsai S-L

Dobbs A

Fitch T

Greenwood J http://orcid.org/0000-0002-6184-0818

Pruessner G

Huang MF

Pakanen V-M

Joly M http://orcid.org/0000-0002-0784-5167

Tschulik K

Hess R

Malic B http://orcid.org/0000-0002-3438-8846

Ralph J

Antanasijević D

Buechley E

Polly D

Watanabe Y http://orcid.org/0000-0001-9731-1769

Yang Y

Xiong C

Kohno Y

Lu G

Quraishi MA

Ledesma-Amaro R

Freeberg T

Stock-Williams C

Lee S H

Ma X

Suharyadi E

Akamatsu F

Zhong L http://orcid.org/0000-0002-8135-2766

Stansby P

Singh R

Aydin N

Salimzadeh S

Serbes G

Leontiadis I

Bento A

LaJeunesse D

Auger-Méthé M

Li X

Wu J

Radke S

Reid C

Yim S http://orcid.org/0000-0003-1322-2713

Wedel M

Mostacci B

Scala F

Heddam S

Hu J

Salah AA

Spitoni G

Goerlitz H http://orcid.org/0000-0002-9677-8073

Subramaniam C

Yin H

Jeffries M

He J

Williams M

Hui C

Stegall S

Beauchamp G

Tofanica B

Pascall A

Smith D http://orcid.org/0000-0002-3427-0936

Poon C

Dougan D http://orcid.org/0000-0001-9313-1904

Bai X

Borges PAV

Wang Z http://orcid.org/0000-0002-8182-2852

Goel A

Proctor M

Hulsey CD http://orcid.org/0000-0002-9653-6728

Daood SS

Huang R

Esteves da Silva JCG

Peirce J http://orcid.org/0000-0002-9504-4342

Su Y

Rossi G http://orcid.org/0000-0002-6540-8467

Zhang J

Anzures G

Mubarak NM

Eriksson A

Storr T

Liu C

Zhuang J

Goswami P

Frediani M

Wilson S http://orcid.org/0000-0001-7841-9643

Ragazzon P

Piantadosi S

Hashmi S

Tanasova M

Govindarajan M

Zhang Z

Ceccarelli G

Kiayias A

Putman N http://orcid.org/0000-0001-8485-7455

Miranda E http://orcid.org/0000-0003-2198-4742

Xie X

Xie Y

Xiang N

Ren J

Narayanan SR

Mondal D

Papathanasiou AG

Sun X

Bonn D

Novas F

Rawlinson N

Mintram K

Voylov D

Li F

Miatto F

Tang L

Voronov A

Mishra S

Horcea-Milcu AI

Parkinson J

Lin Y http://orcid.org/0000-0003-1379-9301

Zhu S-P

Lou W

Cazzola D

Howe L

Li F

Ancona S http://orcid.org/0000-0003-4595-1953

Fay N http://orcid.org/0000-0001-9866-2800

Davis N

Sari E

Soh L

Guo Y http://orcid.org/0000-0002-4985-7553

Weigang Z

Wang H

Rosal R

Hossain M

Di Gioia M

Kukolj T

Shulga D

Bierbach D http://orcid.org/0000-0001-7049-2299

Zhang X

Olsen A http://orcid.org/0000-0003-4398-3126

Sun J

Wang G

Baxendale S

Sircar P

Slattery M

Xu C

Xu Z

Nogaret A

Abdelraheem A http://orcid.org/0000-0001-9633-3333

Chen X

Richard A

Gemeinholzer B

Ratcliff W

Bansal S

Casper M-O

Malik W

Yang S

Wilts B http://orcid.org/0000-0002-2727-7128

Robertsen G

Evran S

Dawel A

Zhang Y-J

Camp A http://orcid.org/0000-0002-3355-4312

Ansari KR

Ellinas C http://orcid.org/0000-0001-5146-6554

Liao Q

Banks P http://orcid.org/0000-0002-4340-6495

Uner O

Lian J http://orcid.org/0000-0001-9784-9876

Anderson K

Zhang H

Babaei B

van der Kooi C http://orcid.org/0000-0003-0613-7633

Heithaus M

Lucas M

Lench H

Wangler T

Ishimoto K http://orcid.org/0000-0003-1900-7643

Ren D

Coote J

Ahmadian R

Xu P http://orcid.org/0000-0002-7747-2423

Guan Y

Jiang B

Peng Y

Ali J

Wang C

Ermentrout G

Trail P

Das S

Schick R

Snowdon C

Dhevalapally R

Conner L

Luis A

Anderson J http://orcid.org/0000-0003-2441-0728

Seedsman R

Danovaro R

Wang B

Liu R

Akin M

Wang W

Wang H

Halliday D

Wu S

Nesbitt S

Zhao K

Zhao Q

Luo K

Choi Y-S

Bi Y

Lin Z http://orcid.org/0000-0002-2729-7583

Bai Q-S

Jani A http://orcid.org/0000-0001-8719-2962

Shen Z

Bonin CA

Brysbaert M

Joel A-C http://orcid.org/0000-0002-7122-3047

McGowan C

Montanari S http://orcid.org/0000-0002-9565-6998

Akturk O

Xue Y

Brown T

Philip J

Hills T

Figueira R

Kaye N

Winternitz J http://orcid.org/0000-0002-1113-9126

Krueger T http://orcid.org/0000-0002-8132-8870

Yang X

Gong P

Murray MB

Zheng Q-H

Jackson A

Bouri E

Ito K

Nigam S

Edwards M

Gibson J

Schott N

Zhou J

Liljenström H

Wilkinson M

Opell B http://orcid.org/0000-0002-1830-0752

Chiu W

Zhou J

Zou Y

Szolnoki A

Ruta M

Malek K

Taylor D

Qiu H

Loderio A

Tan J

Zabet NR

Iosilevskii G http://orcid.org/0000-0002-4114-3214

Strasser R

Agnolin F http://orcid.org/0000-0001-5073-561X

Takamatsu A

Wu M

Luo L

Azhari A

Pei Y

Wang Y

Goletti D

Abdallah H

Hourston DJ

Scapini F http://orcid.org/0000-0001-7229-9572

Cooper B

Scheumann M

Han S

Ahmed A

Kempe V

Bergner L

Elhman S

Laubach SE

Oliveira L

Huang Y

Lindner E

Fernández-Caballero A

Hussein SA

Toczyłowska-Mamińska R

Barucca P

Alessandretti L

Sikavitsas V

Culloch R

Whaley P http://orcid.org/0000-0003-4021-0785

Gladwin T

Huang B

Turkstra L

Good T

Rosenheim J

Pu J

Ramzan M

Porro L

Jakobsen L http://orcid.org/0000-0002-8496-5576

Ahmadi G

Xia M

Shang Y

Moharil SV

Piercy JJB

Farrell M

Botta-Dukát Z http://orcid.org/0000-0002-9544-3474

Lam SK

Wade A

Morand-Ferron J

Kulahci I http://orcid.org/0000-0003-0104-0365

Rader JA

Oikonomou VK

Zhao K

Liang K

Carter O

You B http://orcid.org/0000-0003-1849-0418

Changder S

Liu E http://orcid.org/0000-0002-8624-836X

Gao YZ

Zanin M http://orcid.org/0000-0002-5839-0393

Jiménez S

Fang S

Rahbar N http://orcid.org/0000-0003-0159-0025

Krolikowska A

Hocking D http://orcid.org/0000-0001-6848-1208

Kankala R

Zhuang Z

Ryan EJ

Pritchard D http://orcid.org/0000-0001-7554-3470

Voue M

Mosarof MH

Alais D

Camerlink I http://orcid.org/0000-0002-3427-2210

Li Y

Pisanski K http://orcid.org/0000-0003-0992-2477

Helmy AH

Grace P

Lee C-H

Amos W http://orcid.org/0000-0002-0971-9914

Kusak J

Huang Y

Mobin M

Lee A

Sun B http://orcid.org/0000-0003-3306-656X

Cui X

Alonso D http://orcid.org/0000-0002-8888-1644

Loman ZG

Sillerud L

McGraw K

Rigon A

Keiski MA

Vishal V

Zhaofeng W

Ouyang T

De Buysser K

Ginane C

Yehia AM

Vynnycky M http://orcid.org/0000-0002-8318-1251

Hu C

Shen R

Zhang L

Wang Q

Lee N

Wu K

Athar J

Presley S

Madduri R

Hauert C

Kim G-Y

Mavropoulos GC

Cristofori A

Singh DT

Hall PA

Cai M http://orcid.org/0000-0002-1941-0179

Santos P http://orcid.org/0000-0002-3008-014X

Tryjanowski P http://orcid.org/0000-0002-8358-0797

Singh S

Felsen G

Si K

Bókony V

Pachori RB

Kingsley D

Henzi P http://orcid.org/0000-0001-6175-1674

Druzinsky R http://orcid.org/0000-0002-1572-1316

Gill P

Teng Y

Lu S-G

Jordan A

Pinzari F

Bishop A

Lu Y-H http://orcid.org/0000-0003-1958-2656

Sharma D

Lei W

Zhao N

Henderson D

Choudhury B

Ke Q http://orcid.org/0000-0002-2945-5274

Ozcelik VO

Adams B

Sales L

Klein E

Buckingham G

Nakajima H

Tang D

Mills D

Singh R

Singla A

Yan B

Nakamura N

Mather G http://orcid.org/0000-0002-3102-4828

Kramer R

Rainieri C

Bellisola G

Molleman L http://orcid.org/0000-0003-0184-4240

Robinson K http://orcid.org/0000-0002-6212-9710

Evans J

Nathan A

Wu L

Oliveira U http://orcid.org/0000-0002-4811-757X

Alvarez E

Montomoli F

Jutzeler M

Li Y

Fantuzzi N http://orcid.org/0000-0002-8406-4882

Vilfan A

Birattari M

Reichard M http://orcid.org/0000-0002-9306-0074

Flávia Fernandes K

Schmidt M

Diana G

Shuker D

van de Lagemaat L http://orcid.org/0000-0002-2947-1557

Leopold N

Ganzle M

Wdowin M

Hanley D http://orcid.org/0000-0003-0523-4335

Liu H

Juodkazis S

Ochu E

Shan C

Liu A

Weng W

Usamentiaga R

Grimes D

Manica L

Niu Q

Lucon-Xiccato T

Vosoughi S

Venuti V

Zhao H

Chen S

Fiorelli L

Han G

Aoki K http://orcid.org/0000-0002-8426-4091

Chambers C http://orcid.org/0000-0001-6058-4114

Furia E

Nola IA

Wang K

Sennikov AG

Olberding J http://orcid.org/0000-0001-5426-9986

Wang Z

Tiwary U

Zuo L

Traw B

Piroozfar P

Kaddah MMY

Fischmann A

Schooler J

Grassia P

Sanchez-Muros MJ

Goenezen S http://orcid.org/0000-0002-5051-4371

Forró L

Grasset E http://orcid.org/0000-0002-3062-4733

Gosler A

Leonard J

Smith D

Saarinen J

Ni Y

Parrish C http://orcid.org/0000-0002-1836-6655

Manning A

Temme S

Besley J

Budroni M

Mohammadi SD

Palumbo G

Brand C http://orcid.org/0000-0003-1285-2174

ElBatal HA

Ferrara M

Khan I

Hassanzadeh H

Stone N

Yakovlev V

Lehmann L

Dennis A

Weinzierl R

Stoimenov L

Jayasooriya U

Fujimura K http://orcid.org/0000-0001-6360-7238

Stutz R

Goblirsch M

Lachmann A

Braun E

Brand CM

Song P

Vigilant L

Schultz J

Harel R http://orcid.org/0000-0002-9733-8643

Tatarenkov A http://orcid.org/0000-0002-0516-5862

Gov N http://orcid.org/0000-0001-7774-1139

Archenti A

Pascual-Ezama D

Sato T

Long M

Sugnaseelan S

Jacquin L http://orcid.org/0000-0002-0521-6113

Kendrick K http://orcid.org/0000-0002-0371-5904

Hardy J

Li H

Yang Z

Ahmed I http://orcid.org/0000-0003-3868-2919

Mullon C

Voichita GR http://orcid.org/0000-0003-1143-3111

Tandon P

Gejji SP

Loura A http://orcid.org/0000-0002-4452-4725

Feria Arroyo T

Vuoskoski J

Phan N

Jin F

Ishwar Prasad S

Marzouk MA

Curtis D

Yin W http://orcid.org/0000-0003-4411-1821

Visioli G

Vij J

Okuyama H

Goyens J

Mobley J

Borthwick A

Banerjee J

Davies N http://orcid.org/0000-0002-1740-1412

Qi L

Brennan P

Salvachua D

Bieleke M

Jiang Z

Fernie A

Harbicht A

Nagini S

Boden J

Park Y-Y

Visscher P

Zhang C

Ekiz C

Latosinska J

Sarangapani S

Patel R

Seddon J

Huang S

Stapleton St

Pennock C

Kang J

Horprathum M

Battistel A http://orcid.org/0000-0002-6371-3580

Aydin A

Fisher D http://orcid.org/0000-0002-4444-4450

Criado M

Bar N

Zeng G

Tang Y

Honey R

Ling S

Keiler J

McGrath G

Zhu C

Hilgard J http://orcid.org/0000-0002-7278-4698

Idrus S

Schultz D

Yan J

Chao Y http://orcid.org/0000-0002-8488-2690

Chandra Saha P http://orcid.org/0000-0002-6572-323X

Wu J http://orcid.org/0000-0002-7726-6216

Li M

Besier T

Cai Y

Solomon M

Sanchez-Soto M

Roosen J

Van Kerrebroeck S

Zhang D

Srivastava V

Yamazaki Y

Prater C

Wang D

Ma L

Liu X

Godoy-Diana R http://orcid.org/0000-0001-9561-2699

Smith A

Umoren S

Han S

Andersson J

Krzmarzick M

Wheatley S

Orben R

Zhou D

Kalashnikova M http://orcid.org/0000-0002-7924-8687

Ickert-Bond S

Feng R

Koslover L

Geso M

Welker F

Perez-Porro A

Hirst R

Faria R http://orcid.org/0000-0001-9337-4324

Wu J

Plotkin J

Crespo TM

Van Meeteren M

Kraini M

Chen S

Ohira T

Bernt M

Ribeiro H http://orcid.org/0000-0002-9532-5195

Sobhi M

Osorio D

Li M

Wen H http://orcid.org/0000-0002-2045-729X

Liu Y http://orcid.org/0000-0002-8148-1699

Liu H

Raia P http://orcid.org/0000-0002-4593-8006

Rai P

O'Dea R http://orcid.org/0000-0001-8177-5075

Okura T http://orcid.org/0000-0002-4506-8514

Smolla M http://orcid.org/0000-0001-6367-8765

Arena A

Gewirtz J

Hogan J

Yao Y

Momigliano P

Varotto S

Crevecoeur F

Singh UP

Fricke H

Barzyk T M

Zhou Y

Xu S-A

Dong Y

Corkeron P http://orcid.org/0000-0003-1553-1253

Tran T

Canini L

Zhang Y

Samaniego H http://orcid.org/0000-0002-2485-9827

Proverbio AM

Martin J-M

Saleh T http://orcid.org/0000-0002-3037-5159

Aubriet F

Abbod M

Schulz J

Russell C http://orcid.org/0000-0003-1665-1759

Uzunoglu A

Mahesh MS

Brogdon BN

Li H

Nittono H

Legare C

Wisenden B

Lau G

Dzhelyova M

Huan-Yan X

McRobie A

Du J

Renaud S http://orcid.org/0000-0002-8730-3113

Tambutté S http://orcid.org/0000-0002-0505-6375

Casey D

Jaeger K

Churchland P

Claridge D

Morse DH

Castles A

Holme P

Chen Z http://orcid.org/0000-0002-0161-0236

Orlandi A

Umar Y

Rössner G

Ćosić M

Barts N

Boyd C

Yamagata K

Aitipamula S

Koschella A

Chee M

Fernandes Mendes M

Kiefel M

Hosseinzadeh G

Witte K

Hamburger S

Wijethunga T

Schorr G

Pritsch K

Jover J

Bunsell AR

Sadler P http://orcid.org/0000-0001-9160-1941

Kershenbaum A

Bright J

Drasdo D

Joseph SD

Pogrebna G

Xie Cy

Sanhueza-Chavez C

Clavijo J http://orcid.org/0000-0001-7109-5033

Ros I

Naghizadeh A

Kunwar A

Burgar J

Fraley RC

Straley J

Ruiz-Cooley I

Kalaisselvane A

Vallortigara G http://orcid.org/0000-0001-8192-9062

Akotsen-Mensah C

Briggs K

Heuer H

Renforth P

Leimkuhler B

Arslan R

Bartoš L

Suzuki R

Liu X

Natalello A

Meyerhoff H

Han J

Sabbah S

Kroh A

Imre A

Nardi M

Robertson C

Destgeer G

Wada M

Stein M

Jana N

Peveler W http://orcid.org/0000-0002-9829-2683

Neil T

Lefebvre B http://orcid.org/0000-0002-3803-9176

Takano M

Razi H

Hansford J http://orcid.org/0000-0002-5702-8915

Usher M

van Belzen J

Taylor A http://orcid.org/0000-0003-0071-8306

Dimitri G

Edelaar P http://orcid.org/0000-0003-4649-6366

Ju W

Pluschke G

Nguyen KT http://orcid.org/0000-0003-3934-0371

Boothroyd L

Alaa H

Love G

Manski C http://orcid.org/0000-0001-7260-7686

Trivedi A

Wang K

Nestor A

Tailby C http://orcid.org/0000-0002-1320-5924

Posa D

Guo W-Y http://orcid.org/0000-0003-3589-4537

Chen H

Cheung B

Zhou X

Sun R

Burmeister S http://orcid.org/0000-0001-5612-692X

Butlin R

Seghier M http://orcid.org/0000-0002-1146-8800

Skagenholt M

Calin-Jageman R

DeCaprio A

Jiang L

Williamson M

Drabowicz J

Pomi R

Han PKJ http://orcid.org/0000-0003-0165-1940

Wilson R

Zhang F http://orcid.org/0000-0003-4430-6610

Aizawa M

Windslow R http://orcid.org/0000-0002-1000-5679

Hooker S

Singh G

Longo Jr L

Watts H

Mourao PR

Asai H http://orcid.org/0000-0001-7404-1823

Castaldi E

Hector A

Foulsham T

Carter G http://orcid.org/0000-0001-6933-5501

Panda S

Weber T

Chen G http://orcid.org/0000-0003-1381-7418

Shermolovich YG

Liu C http://orcid.org/0000-0002-5664-0950

Peng L-M

Kelley J http://orcid.org/0000-0002-7731-605X

Qing W

Pang J

Wilshin S

Shalev I

Wei W

Fischhoff B

Ma N

di Virgilio A

Chartier C

Serranti S

Yonemoto Y

Arjmand F

